# Effect of Virtual Reality Gait Training on Participation in Survivors of Subacute Stroke: A Randomized Controlled Trial

**DOI:** 10.1093/ptj/pzab051

**Published:** 2021-02-16

**Authors:** Ilona J M de Rooij, Ingrid G L van de Port, Michiel Punt, Pim J M Abbink-van Moorsel, Michiel Kortsmit, Ruben P A van Eijk, Johanna M A Visser-Meily, Jan-Willem G Meijer

**Affiliations:** 1 Revant Rehabilitation Centres, Breda, the Netherlands; 2 Center of Excellence for Rehabilitation Medicine, UMC Utrecht Brain Center, University Medical Center Utrecht, and De Hoogstraat Rehabilitation, Utrecht, the Netherlands; 3 Research Group Lifestyle and Health, Utrecht University of Applied Sciences, Utrecht, the Netherlands; 4 Department of Neurology, UMC Utrecht Brain Centre, University Medical Centre Utrecht, Utrecht, the Netherlands; 5 Biostatistics & Research Support, Julius Centre for Health Sciences and Primary Care, University Medical Centre Utrecht, Utrecht, the Netherlands; 6 Department of Rehabilitation, Physical Therapy Science & Sports, UMC Utrecht Brain Center, University Medical Center Utrecht, the Netherlands; 7 De Hoogstraat Rehabilitation, Utrecht, the Netherlands

**Keywords:** Gait, Gait Training, Participation, Rehabilitation, Stroke, Virtual Reality

## Abstract

**Objective:**

After stroke, people experience difficulties with walking that lead to restrictions in participation in daily life. The purpose of this study was to examine the effect of virtual reality gait training (VRT) compared to non–virtual reality gait training (non-VRT) on participation in community-living people after stroke.

**Methods:**

In this assessor-blinded, randomized controlled trial with 2 parallel groups, people were included between 2 weeks and 6 months after stroke and randomly assigned to the VRT group or non-VRT group. Participants assigned to the VRT group received training on the Gait Real-time Analysis Interactive Lab (GRAIL), and participants assigned to the non-VRT group received treadmill training and functional gait exercises without virtual reality. Both training interventions consisted of 12 30-minute sessions during 6 weeks. The primary outcome was participation measured with the restrictions subscale of the Utrecht Scale for Evaluation of Rehabilitation-Participation (USER-P) 3 months postintervention. Secondary outcomes included subjective physical functioning, functional mobility, walking ability, dynamic balance, walking activity, fatigue, anxiety and depression, falls efficacy, and quality of life.

**Results:**

Twenty-eight participants were randomly assigned to the VRT group and 27 to the non-VRT group, of whom 25 and 22 attended 75% or more of the training sessions, respectively. No significant differences between the groups were found over time for the USER-P restrictions subscale (1.23; 95% CI = −0.76 to 3.23) or secondary outcome measures. Patients’ experiences with VRT were positive, and no serious adverse events were related to the interventions.

**Conclusions:**

The effect of VRT was not statistically different from non-VRT in improving participation in community-living people after stroke.

**Impact:**

Although outcomes were not statistically different, treadmill-based VRT was a safe and well-tolerated intervention that was positively rated by people after stroke. VR training might, therefore, be a valuable addition to stroke rehabilitation.

**Lay Summary:**

VRT is feasible and was positively experienced by people after stroke. However, VRT was not more effective than non-VRT for improving walking ability and participation after stroke.

## Introduction

Although a substantial number of people after stroke regain the physical capacity to walk without support from others, many still experience difficulties with walking in the community and performing daily life activities.^1^^,^^2^ Previous studies estimated that only approximately 18% to 64% of people after stroke achieve the ability to independently walk in the community without difficulty.^3–6^

An adequate walking ability is necessary to perform daily life activities and to fully participate in the community (eg, work, household, and social activities).^2^^,^^7^ Walking in community environments requires people to adjust their walking to task goals and environmental demands.^8^ This ability is often reduced in people after stroke, and as a result, they experience difficulties with walking far distances; climbing steps, stairs, or inclines; managing terrain irregularity and changes in level; obstacle avoidance; and performing dual tasks during walking.^1^^,^^9^ These difficulties with walking restrict people in performing daily life activities and can limit their participation in the community. Participation is described as “the person’s involvement in life situations,” and problems that one may experience in these life situations are called participation restrictions.^10^ Since walking difficulties can limit participation, improving walking ability is one of the primary goals in stroke rehabilitation.^11^ To achieve an adequate walking ability, it is important to train in different contexts and environments and to include participation as an outcome of rehabilitation.^12^

Virtual reality (VR) is increasingly studied in stroke rehabilitation, including to improve balance and walking function.^13^ Dedicated VR systems with motion capture technology can provide realistic environments in which people can have real-time interaction with objects and events.^14^ A major advantage of VR training is the ability to optimally challenge people in a safe training environment.^15^ While walking in a treadmill-based virtual environment, people have to adapt their walking to unexpected situations (eg, obstacles and perturbations) and can be challenged to perform dual tasks and use their problem-solving abilities. The training can be easily adjusted to the abilities of the patient and the provision of real-time feedback is thought to contribute to motor recovery and to enhance motivation.^13^^,^^16^ Furthermore, principles of motor learning are expected to be applied easily by providing motivating, goal-oriented, varied, and high-intensity training.^13^^,^^15^

Reviews showed favorable effects of various VR systems on balance and walking function in people after stroke.^17–21^ However, previous studies included little evidence about follow-up assessments and lacked outcomes on activities of daily living and participation.^13^^,^^15^^,^^17^ It is therefore unknown whether effects on functional level are translated to real-life daily activity and improvement on participation level.

The primary aim of the present study was to examine the effect of VR gait training (VRT) on participation in community-living for people between 2 weeks and 6 months after stroke. In addition, the effect of VRT on secondary outcomes, including subjective physical functioning, balance and walking ability, and walking activity, was investigated. The second aim was to explore the experiences of people after stroke with VRT, since this training intervention was new for them. Also, we investigated their perception on how this training influenced their walking ability and participation. We hypothesized that treadmill-based VRT is a safe training intervention that is superior to a non-VRT consisting of conventional treadmill training and functional gait exercises.

## Methods

### Study Design and Participants

We conducted an assessor-blinded, randomized controlled trial with 2 parallel groups to investigate the effect of VRT on participation in people after stroke. Participants were equally allocated to the VRT group or non-VRT group. The full study protocol of this study, called ViRTAS (Virtual Reality Training After Stroke), has been published previously.^22^ No changes were made to the study design or eligibility criteria after study commencement.

Participants were primarily recruited by their physician or physical therapist at the rehabilitation center. In addition, participants were recruited at the neurology department of the local hospital and physical therapy and general practices in the area. For inclusion, potential participants had to meet the following criteria: (1) diagnosed with stroke according to the World Health Organization definition,^23^ (2) time since stroke between 2 weeks and 6 months, (3) ability to walk without physical assistance for balance and coordination (Functional Ambulation Category ≥ 3),^24^ (4) experiencing self-perceived constraints with walking in daily life, (5) living in the community, and (6) age 18 to 80 years. Exclusion criteria were insufficient cognitive skills or understanding of the Dutch language to reliably answer simple questions; severe visual impairments, severe forms of ataxia, or uncontrolled epileptic seizures; and orthopedic disorders or other comorbidities that limited current walking ability. All participants provided written informed consent.

### Procedures

Participants were randomly assigned to the VRT or non-VRT group by an independent expert not involved in the recruitment, intervention, or assessments. Randomization was performed using sealed, opaque envelopes that contained a card stipulating to which group the participant was allocated.

Both groups received a training intervention that consisted of 2 30-minute sessions per week for 6 weeks (12 sessions). Assessments were performed at baseline (T0), postintervention (T1, 6 weeks), and follow-up (T2, 3 months postintervention) by a researcher blinded to group allocation (I.d.R.). The participants and intervention therapists could not be blinded to group allocation because of the nature of the intervention. Training sessions and assessments were conducted in the rehabilitation center, Revant Rehabilitation Centres, Breda, the Netherlands. All adverse events were registered and serious adverse events were reported to the medical ethics review committee.

### Interventions

Experienced physical therapists conducted the VRT and non-VRT intervention and adapted each training session to the abilities and needs of the participants. Based on clinical expertise, the physical therapists regulated the intensity of the training, decided the amount of progression, and ensured safety and quality of movement during the training. During the 6 weeks of training difficulty was increased. The intensity of each training session was monitored by scoring the rate of perceived exertion with Borg’s Category-Ratio Scale of Perceived Exertion (range, 0–10) and measuring number of steps taken with a pedometer (Digi-Walker SW-200; Yamax Corp, Tokyo, Japan).

#### Intervention Group

The VRT group trained on the Gait Real-time Analysis Interactive Lab (GRAIL; Motekforce Link, Amsterdam, the Netherlands). The GRAIL consists of an instrumented dual-belt treadmill combined with a motion-capture system (Vicon Motion Systems, Oxford, UK) and a 180° semi-cylindrical screen for the projection of synchronized 3-dimensional environments. To create a safe environment, participants wore a harness without providing weight support. Various rehabilitative applications (VR environments) with specific rehabilitation goals were available (eg, to train reactive balance, maneuverability, or dual tasks). Difficulty level could be further modified within the applications by adjusting multiple training options: duration, treadmill speed, pitch and sway of the treadmill, belt acceleration or deceleration, amount of simultaneous tasks, frequency and position of environmental constrains (eg, obstacles), and the amount and type of real-time feedback. VRT was conducted by specialized therapists who are trained to work with the GRAIL**.** The therapist chose, based on the therapeutic goals, which applications were used during a training session and could easily adapt the difficulty level to individual abilities of the participants.

#### Comparison Group

The non-VRT intervention combined 2 commonly used interventions to improve walking ability: (1) conventional treadmill training (10–15 minutes) and (2) functional gait exercises (15 minutes). Duration, speed, and/or incline of the treadmill were increased and support from handrails was decreased to make the intervention progressive. The functional gait exercises included 6 directional exercises: (1) tapping or stepping up and down a step, (2) walking and picking up various objects from the ground, (3) walking on nonlevel surface, (4) walking a slalom, (5) stepping in hoops, and (6) stepping over a stick that is fixed between 2 pylons. The exercises were based on the exercises used in the FIT-Stroke trial.^25^ The therapist chose which exercises were conducted during the different training sessions. Exercises were individualized by adapting amount of repetitions, distance, height, variation, and amount of dual tasks, for example.

### Outcome Measures

#### Primary Outcome

The primary outcome measure was participation as measured with the restrictions subscale of the Utrecht Scale for Evaluation of Rehabilitation-Participation (USER-P) using all available data up to 3 months postintervention.^26^ The restrictions subscale of the USER-P assesses the experienced participation restrictions in daily life and consists of 11 items. The total score ranges from 0 to 100 with higher scores indicating less experienced restrictions.^27^

#### Secondary Outcomes

Secondary outcome measures included the frequency and satisfaction subscales of the USER-P. Other outcomes were questionnaires regarding subjective physical functioning (Stroke Impact Scale-16), fatigue (Fatigue Severity Scale), anxiety and depression (Hospital Anxiety and Depression Scale), falls efficacy (Falls Efficacy Scale International), and quality of life (Stroke Specific Quality of Life Scale). Performance tests measured functional mobility (Timed “Up & Go” Test), walking ability (6-minute walking test), and dynamic balance (Mini Balance Evaluation Systems Test [Mini-BESTest]). In addition, daily-life walking activity was measured with a triaxial accelerometer (hardware: DynaPort MM, McRoberts BV, the Hague, the Netherlands) during 5 consecutive days.

Detailed descriptions of all secondary outcomes are available in the original protocol.^22^ The Mini-BESTest was added to the original protocol to assesses dynamic balance.^28^ The test measures 4 underlying systems for balance control and consists of 14 items. The total score ranges from 0 to 28 points. A higher total score indicates better balance performance.^29^

Walking activity data from the accelerometer was analyzed with a stroke-specific algorithm for gait detection and quantification in Matlab (The MathWorks Inc, Natick, MA, USA).^30^ Walking activity was expressed as total number of steps a day, total duration of walking activity per day (minutes), and step frequency (number of steps per minute of walking activity). The algorithm detected walking activity with a minimum length of 8 seconds or a multiple thereof. The total duration of walking activity was calculated as the sum of all 8-second walking bouts per day. Step frequency was calculated by dividing the total number of steps by the total duration of walking activity.^31^ The walking activity values were averaged over the days on which participants wore the accelerometer for at least 8 hours. Participants had to wear the accelerometer for at least 3 days and had to walk, on average, at least 5 minutes per day to be included in the analysis.^31^

In addition, patients’ experiences with VRT were explored using semi-structured interviews. The semi-structured interviews were conducted by 2 independent researchers at the end of the 6-week training period. In a face-to-face session, the interviewer asked participants about their experiences with VRT (including advantages or disadvantages) and their perception of how the VRT intervention influenced their walking ability and participation. Interviews were audio-recorded, transcribed verbatim, and analyzed thematically.^32^ Themes and codes were analyzed and summarized in NVivo 12 (QRS International, Burlington, MA, USA).

### Data Analysis

The medical ethics review committee approved a sample size of 56 participants. This sample size was corrected when reviewers of the protocol article pointed out a miscalculation. In the revised calculation, a total of 50 (25 per group) people after stroke would be required to detect a mean difference of 15% (15 points, SD = 17.9) on the USER-P restrictions subscale with 80% power and a 2-sided α of 5%, while accounting for an estimated dropout rate of 10% to 20%.^22^ However, because randomization was already arranged, we aimed to recruit as close to 56 participants in the time available.

We visually checked normality for all continuous outcome measures. Right-skewed outcome variables involving time were log-transformed before the analyses. The analysis was performed based on a modified intention-to-treat principle including all participants who attended at least one follow-up measurement. The effectiveness of VRT on the primary outcome measure, USER-P restrictions subscale, was analyzed using an analysis of covariance linear mixed-effects model. The fixed part of the model included an effect for baseline USER-P restrictions, time in months, group assignment (VRT or non-VRT), and the interaction between time and group. The random part of the model contained a random intercept per individual. Treatment effectiveness was determined by evaluating the significance of the time by group interaction (ie, is the effect over time different for participants allocated to VRT or non-VRT). For the secondary outcome variables, comparable linear mixed-effects models were used with correction for baseline scores of the respective outcome variables.

Furthermore, 2 sensitivity analyses were conducted for the primary outcome. In the first sensitivity analysis the interaction between baseline USER-P restrictions and time was added as additional adjustment variable. In the second sensitivity analysis, we adjusted our primary model for time since stroke due to a potential imbalance between groups. In addition, we performed a per-protocol analysis for the primary outcome in which only data of participants who attended at least 75% of the training sessions were included. Analyses were performed in SPSS version 25 (IBM Corp, Armonk, NY, USA) and results were considered significant when *P* values were less than .05.

### Role of the Funding Source

The funder played no role in the design, conduct, or reporting of the study.

## Results

Between April 19, 2017 and July 26, 2019, a total of 55 participants were recruited as planned, of which 28 were randomly assigned to the VRT group (18 men, 10 women) and 27 to the non-VRT group (19 men, 8 women). Three participants in the non-VRT group were excluded from the modified intent-to-treat analysis because they did not complete any follow-up assessment. One did not attend any assessment and did not start the intervention (reason unknown because of nonresponse), and 2 other participants dropped out before the postintervention assessment because of recurrent stroke. Fifty-two participants were included in the modified intention-to-treat analysis (Figure). Of these 52 participants, 2 discontinued the VRT intervention because of unexpected abdominal surgery and low physical condition, and 1 the non-VRT intervention because of difficulties with transport.

**Figure:**
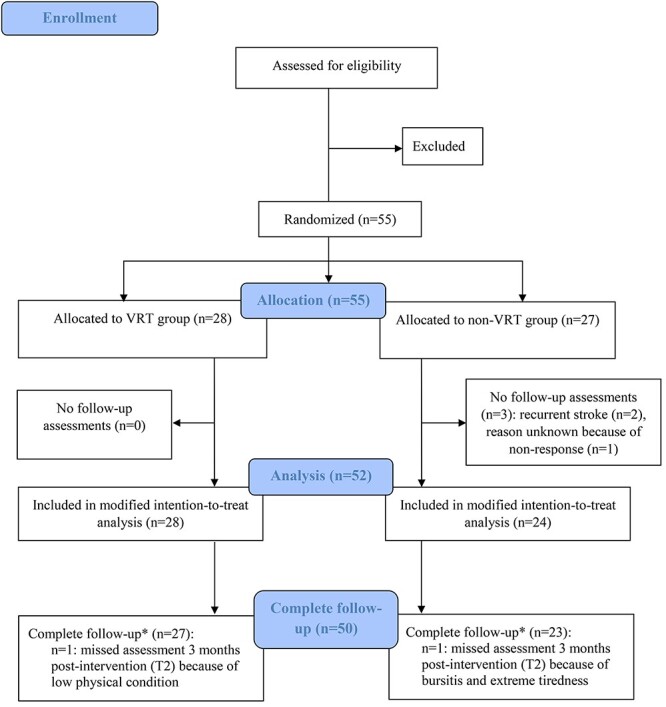
Flow diagram of participants ^*^Complete follow-up included both the postintervention assessment (T1, 6 wk) and the follow-up assessment (T2, 3 mo postintervention).

Table 1 shows the baseline demographic and clinical characteristics of the participants. Baseline characteristics of the groups were similar, except for time since stroke which was higher in the VRT group (84 vs 66 days). Twenty-four participants in the VRT and 23 participants in the non-VRT group received additional outpatient rehabilitation (n = 39) or physical therapy and occupational therapy in primary care (n = 8) during the intervention period. Medication use and amount of comorbidities were comparable in both groups.

**Table 1:** Demographic and Clinical Characteristics of 52 Participants in Virtual Reality Gait Training (VRT) and Non-VRT Groups at Baseline^a^

**Variable**	**VRT Group (n = 28)**	**Non-VRT Group (n = 24)**	**Overall Group (n = 52)**
Demographic			
Age at baseline, y*^b^*	65 (57–70)	61 (53–71)	63 (55–70)
Height, m*^b^*	1.75 (1.67–1.80)	1.76 (1.67–1.85)	1.75 (1.67–1.80)
Weight, kg*^b^*	77 (69–88)	75 (68–88)	76 (69–88)
Sex			
Men	18 (64.3)	18 (75.0)	36 (69.2)
Women	10 (35.7)	6 (25.0)	16 (30.8)
Partner			
Yes	23 (82.1)	20 (83.3)	43 (82.7)
No	5 (17.9)	4 (16.7)	9 (17.3)
Living situation			
Alone	5 (17.9)	4 (16.7)	9 (17.3)
With partner	23 (82.1)	19 (79.2)	42 (80.8)
With other family members	0 (0.0)	1 (4.2)	1 (1.9)
Region of residence*^c^*			
Rural or small town	10 (35.7)	4 (16.7)	14 (26.9)
Small urban	11 (39.3)	17 (70.8)	28 (53.8)
Large urban	7 (25.0)	3 (12.5)	10 (19.2)
Injury-related clinical			
Time since stroke at baseline, d*^b^*	84 (69–110)	66 (51–103)	76 (54–110)
Type of stroke			
Ischemic	24 (85.7)	20 (83.3)	44 (84.6)
Hemorrhagic	4 (14.3)	4 (16.7)	8 (15.4)
Site of stroke			
Left hemisphere	15 (53.6)	12 (50.0)	27 (51.9)
Right hemisphere	10 (35.7)	10 (41.7)	20 (38.5)
Brainstem	3 (10.7)	2 (8.3)	5 (9.6)
Previous stroke			
Yes	4 (14.3)	2 (8.3)	6 (11.5)
No	24 (85.7)	22 (91.7)	46 (88.5)
Functional ambulation category score			
3	3 (10.7)	0 (0.0)	3 (5.8)
4	4 (14.3)	9 (37.5)	13 (25.0)
5	21 (75.0)	15 (62.5)	36 (69.2)
Use of mobility aids outdoors, yes	14 (50.0)	11 (45.8)	25 (48.1)
Single-point cane or crutch	3 (10.7)	5 (20.8)	8 (15.4)
Rollator	8 (28.6)	7 (29.2)	15 (28.8)
4-point stick	2 (7.1)	0 (0.0)	2 (3.8)
Wheelchair	1 (3.6)	1 (4.2)	2 (3.8)
Mobility scooter	2 (7.1)	1 (4.2)	3 (5.8)
Use of ankle-foot orthoses			
Yes	5 (17.9)	8 (33.3)	13 (25.0)
No	23 (82.1)	16 (66.7)	39 (75.0)
Therapy related			
Duration of gait-related therapies parallel to study intervention, h*^b^*			
Physical therapy	5.8 (4.0–7.6)	5.5 (4.5–6.0)	5.5 (4.3–6.0)
Occupational therapy	5.5 (2.5–6.0)	4.5 (2.0–5.5)	5.0 (2.4–6.0)
Medical fitness	10.0 (5.0–11.3)	10.0 (2.0–11.3)	10.0 (5.0–11.0)
Psychomotor therapy	3.5 (3.0–10.0)	8.0 (4.5–9.0)	7.0 (3.0–9.8)

^*a*^Values are reported as number (%) of participants unless stated otherwise. VRT = virtual reality gait training.

^*b*^Reported as median (25th–75th percentiles).

^*c*^Rural or small town = population of less than 10,000; small urban = population of 10,000 to 99,999; large urban = population greater than or equal to 100,000.

### Training Characteristics and Patients’ Experiences

In total, 89.3% of the participants in the VRT (n = 25) and 91.7% of the participants in the non-VRT group (n = 22) attended 75% or more of the training sessions. Intensity of the training sessions was comparable for both groups as shown with a mean number of steps of 1305 (463) in the VRT (n = 25) and 1271 (432) in the non-VRT group (n = 22, *P* = .80). The mean change in the Category-Ratio Scale of Perceived Exertion between start and end of a training session did also not significantly differ between the groups (VRT: 2.91 [1.15], non-VRT: 3.21 [1.39], *P* = .43). No serious adverse events were reported related to the study interventions. Adverse events reported during the VRT sessions included 3 near falls, dizziness (1 participant), fatigue (3 participants), and muscle stiffness or pain in the legs (4 participants). During the non-VRT sessions, adverse events were 3 near falls, headache, increased clonus in the foot, breathing difficulty (1 participant), dizziness (4 participants), fatigue (4 participants), and pain in the legs or back (3 participants). Some adverse events led to more rest breaks or premature stop of a training session but none of the events led to discontinuation of the intervention.

Results from 16 semi-structured interviews showed that participants experienced the VRT intervention as enjoyable, challenging, and intensive. The most frequently mentioned advantages included the safe training environment and the opportunities to train dual tasks, reaction time, obstacle avoidance, and reactive balance. In addition, participants appreciated the variation in exercises, the game elements, and the high intensity of the training. Experienced disadvantages were awareness of the safety harness, lack of real objects to step over, flash effects on the screen, and technical deficits of the VR system. The majority of the participants stated that VRT positively influenced their walking ability. Most mentioned effects that participants experienced were improved balance ability, improved ability to perform dual tasks, improved ability to take steps and to walk on irregular surfaces, improved reaction time, and a higher walking speed. In addition, some participants mentioned that walking was experienced with more ease and movement automaticity after the VRT intervention. Perceived effects on daily life participation were improved confidence during walking, improved ability to cope with busy environments and stimuli, improved walking endurance, and less difficulty with self-care and household chores. Despite the fact that participants also received other therapies, they felt that VRT definitely contributed to their experienced improvements.

### Efficacy Outcomes

Table 2 shows outcomes at baseline (T0), postintervention (T1), and follow-up (T2) for both groups. Baseline scores of the groups were similar for all outcome measures. Results for all participants together, irrespective of group allocation, showed significant improvements in participation and dynamic balance over time as quantified by the USER-P restrictions subscale (monthly increase of 2.53 points; 95% CI = 1.52 to 3.54, *P* < .001), the USER-P frequency subscale (1.05, 95% CI = 0.50 to 1.61, *P* < .001), and the Mini-BESTest score (0.62, 95% CI = 0.33 to 0.91, *P* < .001).

**Table 2:** Outcomes at Baseline, Postintervention, and at Follow-up Assessment (N = 52)^a^

Outcome	**VRT group**			**Non-VRT group**		
	**Baseline (n = 28)**	**Postintervention (n = 28)**	**3-mo Follow-up (n = 27)**	**Baseline (n = 24)**	**Postintervention (n = 24)**	**3-mo Follow-up (n = 23)**
Primary						
USER-P restrictions	59.70 (19.04)	71.58 (17.43)	80.76 (15.86)	63.92 (15.06)	75.72 (15.20)	81.62 (15.75)
Secondary						
USER-P frequency	27.50 (8.19)	32.93 (7.74)	35.82 (7.71)	26.49 (11.22)	32.53 (7.40)	35.56 (9.04)
USER-P satisfaction	56.76 (16.77)	69.18 (14.74)	72.47 (15.93)	57.47 (18.02)	69.14 (17.18)	72.92 (17.53)
6-MWT, m	359.54 (124.26)	408.19 (125.34)*^b^*	419.22 (129.30)	357.79 (104.13)	428.45 (110.37)*^c^*	428.66 (103.37)*^c^*
TUG, s, geometric mean (95% CI)	12.41 (10.52 to 14.64)	10.76 (9.18 to 12.62)	10.37 (8.74 to 12.30)	11.85 (10.41 to 13.49)	9.94 (8.79 to 11.24)*^d^*	9.88 (8.89 to 10.99)
Mini-BESTest	17.56 (5.88)*^e^*	19.93 (6.84)*^f^*	21.67 (6.79)*^f^*	20.08 (5.07)*^g^*	22.33 (4.10)*^g^*	24.42 (2.02)*^g^*
FES-I	25.57 (9.57)	20.96 (4.75)	20.67 (5.68)	23.54 (5.12)	20.75 (5.56)	20.78 (6.16)
FSS	5.17 (1.41)	4.40 (1.62)	3.97 (1.65)	4.75 (1.56)	4.46 (1.17)	4.26 (1.66)
SIS-16	80.22 (12.31)	87.09 (9.46)	89.03 (11.13)	79.41 (9.71)	91.35 (7.52)	90.80 (7.86)
HADS anxiety	4.36 (3.70)	3.50 (3.18)	3.33 (3.46)	4.00 (2.80)	3.33 (2.68)	2.39 (2.50)
HADS depression	4.39 (3.35)	4.04 (3.49)	3.56 (3.51)	3.54 (2.28)	2.83 (2.16)	2.65 (2.50)
SS-QOL	3.87 (0.71)*^h^*	4.21 (0.56)*^i^*	4.36 (0.52)*^j^*	3.94 (0.56)	4.36 (0.50)	4.35 (0.55)
No. of steps/d	3643 (1859)*^k^*	3761 (1995)*^c^*	3870 (2417)*^i^*	4855 (2967)*^c^*	4588 (2745)*^h^*	4844 (2587)*^l^*
Walking duration, min/d	39.38 (19.46)*^k^*	39.70 (19.95)*^c^*	41.71 (25.59)*^i^*	52.50 (29.37)*^c^*	47.88 (24.34)*^h^*	51.12 (22.80)*^l^*
Step frequency, steps/min	92.52 (9.25)*^k^*	93.99 (10.52)*^c^*	92.78 (11.16)*^i^*	91.29 (10.80)*^c^*	94.38 (13.69)*^h^*	92.61 (13.92)*^l^*
Accelerometer wearing time, h	17.75 (3.60)*^k^*	17.94 (3.78)*^c^*	17.77 (3.89)*^i^*	18.61 (2.56)*^c^*	19.09 (3.21)*^h^*	18.83 (2.86)*^l^*

^*a*^Values are reported as mean (SD) unless stated otherwise. 6-MWT = 6-min walking test; FES-I = Falls Efficacy Scale International, scored from 16 to 64; FSS = Fatigue Severity Scale, scored from 1 to 7; HADS = Hospital Anxiety and Depression Scale, scored from 0 to 21; Mini-BESTest = Mini Balance Evaluation Systems Test, scored from 0 to 28; SIS-16 = Stroke Impact Scale-16, scored from 0 to 100; SS-QOL = Stroke Specific Quality of Life Scale, scored from 1 to 5; TUG = Timed “Up & Go” Test; USER-P = Utrecht Scale for Evaluation of Rehabilitation-Participation, scored from 0 to 100; VRT = virtual reality gait training.

^*b*^n = 27.

^*c*^n = 22.

^*d*^n = 23.

^*e*^n = 16.

^*f*^n = 15.

^*g*^n = 12.

^*h*^n = 21.

^*i*^n = 24.

^*j*^n = 26.

^*k*^n = 25.

^*l*^n = 19.

We found no statistically significant difference in USER-P restriction score between the VRT and non-VRT group over time. The VRT group increased by 3.11 points per month vs 1.88 points per month in the non-VRT group, resulting in a mean difference of 1.23 (95% CI = −0.76 to 3.23, *P* = .22). Although not significant, the USER-P restrictions score improved 1.23 points more per month in the VRT group compared with the non-VRT group (Tab. 3). The 2 sensitivity analyses with the interaction between the baseline USER-P restrictions variable and time and with time since stroke did not lead to substantially different results. Also, the per-protocol analysis, including participants who attended at least 75% of the training sessions (n = 47), yielded comparable results (mean difference = 1.47; 95% CI = −0.60 to 3.53, *P* = .158).

**Table 3:** Monthly Changes and *P* Values Per Outcome (Modified Intention-to-Treat Analysis)^a^

**Outcome**	**Monthly Change in VRT Group (95% CI) (n = 28)**	**Monthly Change in non-VRT Group (95% CI) (n = 24)**	**Mean Slope Difference (95% CI)**	***P***
Primary				
USER-P restrictions	3.11 (1.74 to 4.49)	1.88 (0.44 to 3.33)	1.23 (−0.76 to 3.23)	.221
Secondary				
USER-P frequency	1.05 (0.28 to 1.82)	1.06 (0.25 to 1.86)	−0.01 (−1.12 to 1.11)	.992
USER-P satisfaction	1.10 (−0.57 to 2.78)	1.14 (−0.62 to 2.90)	−0.04 (−2.46 to 2.39)	.976
6-MWT, m	3.65 (0.28 to 7.01)*^b^*	0.00 (−3.60 to 3.61)*^c^*	3.64 (−1.29 to 8.57)	.144
TUG log transformed	−0.01 (−0.02 to 0.01)	−0.00 (−0.02 to 0.01)*^d^*	−0.01 (−0.03 to 0.01)	.453
Mini-BESTest	0.57 (0.19 to 0.96)*^e^*	0.68 (0.24 to 1.11)*^f^*	−0.10 (−0.69 to 0.48)	.721
FES-I	−0.06 (−0.61 to 0.49)	0.08 (−0.50 to 0.65)	−0.13 (−0.93 to 0.67)	.740
FSS	−0.13 (−0.30 to 0.04)	−0.06 (−0.23 to 0.12)	−0.08 (−0.33 to 0.17)	.523
SIS-16	0.66 (−0.12 to 1.45)	−0.23 (−1.05 to 0.60)	0.89 (−0.25 to 2.03)	.123
HADS anxiety	−0.02 (−0.26 to 0.23)	−0.27 (−0.53 to −0.02)	0.26 (−0.10 to 0.61)	.148
HADS depression	−0.15 (−0.45 to 0.15)	−0.02 (−0.34 to 0.29)	−0.13 (−0.57 to 0.31)	.563
SS-QOL	0.04 (−0.01 to 0.09)*^g^*	−0.00 (−0.05 to 0.04)	0.04 (−0.03 to 0.11)	.204
No. of steps/d	133.84 (−137.22 to 404.90)*^d^*	122.26 (−157.12 to 401.64)*^h^*	11.58 (−377.60 to 400.76)	.952
Walking duration, min/d	1.69 (−0.90 to 4.27)*^d^*	1.26 (−1.41 to 3.93)*^h^*	0.43 (−3.28 to 4.14)	.816
Step frequency, steps/min	−0.42 (−1.94 to 1.11)*^d^*	−0.37 (−1.94 to 1.20)*^h^*	−0.05 (−2.24 to 2.14)	.964
Accelerometer wearing time, h	0.05 (−0.22 to 0.31)*^d^*	−0.16 (−0.44 to 0.11)*^h^*	0.21 (−0.17 to 0.60)	.265

^*a*^FES-I = Falls Efficacy Scale International, scored from 16 to 64; 6-MWT = 6-min walking test; FSS = Fatigue Severity Scale, scored from 1 to 7; HADS = Hospital Anxiety and Depression Scale, scored from 0 to 21; Mini-BESTest = Mini Balance Evaluation Systems Test, scored from 0 to 28; SIS-16 = Stroke Impact Scale-16, scored from 0 to 100; SS-QOL = Stroke Specific Quality of Life Scale, scored from 1 to 5; TUG = Timed “Up & Go” Test; USER-P = Utrecht Scale for Evaluation of Rehabilitation-Participation, scored from 0 to 100; VRT = virtual reality gait training.

^*b*^n = 27.

^*c*^n = 22.

^*d*^n = 23.

^*e*^n = 15.

^*f*^n = 12.

^*g*^n = 24.

^*h*^n = 21.

Secondary outcome measures did not reveal any statistically significant differences between the VRT and non-VRT group over time (Tab. 3).

## Discussion

This study showed that VRT is a safe and well-received intervention. The positive patient experiences from the semi-structured interviews were not, however, supported by statistically significant between-group differences in quantitative outcome measures. The effect of VRT on participation was statistically not different from non-VRT in community-living people after stroke. Secondary outcome measures, including subjective physical functioning, balance and walking ability, walking activity, and falls efficacy, did also not show significant greater improvements in the VRT group compared to the non-VRT group.

To our knowledge, this is the first study that investigated the effect of a treadmill-based VRT on the level of participation in people after stroke. Participation improved both in the VRT and non-VRT groups. The VRT group improved 3.11 points per month on the USER-P restrictions subscale versus 1.88 points per month in the non-VRT group. A large study about the course of quality of life and participation after stroke found a mean increase of 5.04 points on the USER-P restrictions subscale between 2 and 6 months after stroke.^33^ This represents a mean increase of 1.26 points per month. Compared with this previous study, the improvement in participation in the VRT group was more than twice as great, while improvement in the non-VRT group was more comparable. From a noninferiority perspective, the lower confidence bound of the mean difference between the VRT and non-VRT group is likely irrelevant and may indicate that the VRT intervention is possibly noninferior to the non-VRT intervention.

There are several possible explanations for the lack of significant between-group differences. First, the potential difference in participation might be influenced by spontaneous neurologic recovery. Previous studies showed that spontaneous recovery is an important contributor to overall functional and neurologic recovery and mainly takes place within the first 3 months after stroke.^34^^,^^35^ Participants in the VRT group had, on average, a longer period since their stroke and may therefore be less prone to spontaneous recovery compared to the non-VRT group. However, the sensitivity analysis showed no confounding effect of time since stroke. Second, the contrast between the VRT and non-VRT intervention might have been too small to find significant differences.^36^^,^^37^ Although the training environment clearly differed, both interventions were intended to provide functional, personalized, and progressive training. A third explanation may be that we did not use the possibility to personalize the VRT intervention optimally. We did not specifically measure underlying walking impairments at the functional level nor did we assess achievement of patient-specific rehabilitation goals related to participation restrictions. There is some evidence that VRT focusing on specific aspects of walking function, like reactive balance, can result in a more stable gait.^38^ Measurement of underlying walking impairments and individual rehabilitation goals would have given the opportunity to make the training even more personalized and could have given more insight into which patients are most likely to benefit from VRT.

Although effects were not significantly different, participation improved more in the VRT group. VRT was provided using an immersive and interactive VR system with motion-capture technology that was designed for rehabilitation. This specific VR intervention was thought to promote important principles of motor learning by providing high-intensity training with task variation and real-time feedback.^13^^,^^39^ These principles were confirmed in semi-structured interviews with participants after stroke. According to the participants, most advantageous training characteristics were the safe training environment, the high intensity, and the opportunities to train dual tasks, reaction time, and balance perturbations during walking. The safe training environment, together with the adjustable difficulty levels, allowed therapists to optimally challenge participants. In this way, VRT gave people after stroke more insight in their possibilities and limitations and improved confidence in their walking abilities. In addition, dual tasks and reactive balance could be easily and safely trained. The ability to practice tasks that are unsafe to practice in the real world is one of the major benefits of VR.^15^ Furthermore, participants liked the game elements and variation in VR environments, which made the training more enjoyable and promoted implicit learning. Implicit learning is thought to improve movement automaticity and dual-task performance.^40^ The experiences of enjoyment and immersion are in line with a study by Cano Porras et al^41^ that investigated clinical experiences with VRT in a rehabilitation population. They found that the most positive feedback obtained from patients included topics such as enjoyment, immersion, clarity, easiness of the VR system, and lack of discomfort or adverse events.

In addition, results from the semi-structured interviews gave more insight into patients’ perception of how VRT influenced their walking ability and participation. Participants suggested that besides walking ability, VRT promoted improvements in cognitive function (reacting quickly and dealing with busy environments and stimuli), confidence during walking, and movement automaticity. A previous study by Fishbein et al^42^ found higher self-confidence when walking in the community after dual-task treadmill training using VR, suggesting that VRT can positively target self-confidence. Regarding cognitive function, systematic reviews showed the potential of VR for improving cognitive function in people after stroke, but until now results have yielded inconclusive evidence about the effectiveness of VR training on improving cognition.^43^^,^^44^ The mentioned improvements in this domain suggest that proper measures of cognition should be included in future studies, even in a seemingly well-functioning population.

The present study had some limitations. The first limitation was a large variability in experienced participation restrictions on the USER-P at baseline. This suggests that the sample size might have been too small to determine between-group differences in the mixed-model analyses. The positive trend for greater improvement of participation in the VRT group suggests that with a higher sample size significant between-group differences might have been found. A second limitation was that the same therapists performed both the VRT and non-VRT intervention. Because the therapists knew the content and training possibilities of both interventions, they may have had more difficulty completely separating the content of both interventions and concealing whether they preferred one intervention to the other.

## Conclusion

The effect of VRT was not statistically different from the effect of non-VRT on participation in community-living people after stroke. Both interventions, however, contribute to improvement in participation and can be applied in stroke rehabilitation taking into account the individual rehabilitation goals or patient preference. Although not statistically different, participation was shown to improve more in the VRT group. VRT was positively rated by people after stroke and was shown to be a well-tolerated and clinically practicable intervention, including appropriate levels of adherence and limited adverse events. These results suggest that treadmill-based VRT can be a valuable addition to stroke rehabilitation. Future studies should further explore the cost-effectiveness and added value of VRT for specific rehabilitation goals.

## Clinical Trial Registration

This study was registered in the Netherlands National Trial Register (identifier NTR6215).

## References

[1] Mayo NE, Wood-Dauphinee S, Côté R, Durcan L, Carlton J. Activity, participation, and quality of life 6 months poststroke. Arch Phys Med Rehabil. 2002;83:1035–1042. 12161823 10.1053/apmr.2002.33984

[2] de Rooij IJM, van de Port IGL, van der Heijden LLM, Meijer JG, Visser-Meily JMA. Perceived barriers and facilitators for gait-related participation in people after stroke: from a patients’ perspective. Physiother Theory Pract. 2019;20:1–9. 10.1080/09593985.2019.1698085 31793365

[3] Rosa MC, Marques A, Demain S, Metcalf CD. Fast gait speed and self-perceived balance as valid predictors and discriminators of independent community walking at 6 months post-stroke—a preliminary study. Disabil Rehabil. 2015;37:129–134. 24754638 10.3109/09638288.2014.911969

[4] Jørgensen HS, Nakayama H, Raaschou HO, Olsen TS. Recovery of walking function in stroke patients: the Copenhagen Stroke Study. Arch Phys Med Rehabil. 1995;76:27–32. 7811170 10.1016/s0003-9993(95)80038-7

[5] Viosca E, Lafuente R, Martínez JL, Almagro PL, Gracia A, González C. Walking recovery after an acute stroke: assessment with a new functional classification and the Barthel index. Arch Phys Med Rehabil. 2005;86:1239–1244. 15954066 10.1016/j.apmr.2004.11.015

[6] Perry J, Garrett M, Gronley JK, Mulroy SJ. Classification of walking handicap in the stroke population. Stroke. 1995;26:982–989. 7762050 10.1161/01.str.26.6.982

[7] Robinson CA, Shumway-Cook A, Matsuda PN, Ciol MA. Understanding physical factors associated with participation in community ambulation following stroke. Disabil Rehabil. 2011;33:1033–1042. 20923316 10.3109/09638288.2010.520803

[8] Balasubramanian CK, Clark DJ, Fox EJ. Walking adaptability after a stroke and its assessment in clinical settings. Stroke Res Treat. 2014;2014:591013. 25254140 10.1155/2014/591013 PMC4164852

[9] Plummer-D’Amato P, Altmann LJ, Saracino D, Fox E, Behrman AL, Marsiske M. Interactions between cognitive tasks and gait after stroke: a dual task study. Gait Posture. 2008;27:683–688. 17945497 10.1016/j.gaitpost.2007.09.001 PMC2913384

[10] World Health Organization . *International Classification of Functioning,*Disability and Health: ICF. Geneva, Switzerland: WHO; 2001.

[11] Craig LE, Wu O, Bernhardt J, Langhorne P. Predictors of poststroke mobility: systematic review. Int J Stroke. 2011;6:321–327. 21745343 10.1111/j.1747-4949.2011.00621.x

[12] Engel-Yeger B, Tse T, Josman N, Baum C, Carey LM. Scoping review: the trajectory of recovery of participation outcomes following stroke. Behav Neurol. 2018;2018:5472018. 30271506 10.1155/2018/5472018 PMC6151208

[13] Levin MF . What is the potential of virtual reality for post-stroke sensorimotor rehabilitation? Expert Rev Neurother. 2020;20:195–197. 32050825 10.1080/14737175.2020.1727741

[14] Perez-Marcos D . Virtual reality experiences, embodiment, videogames and their dimensions in neurorehabilitation. J Neuroeng Rehabil. 2018;15:113. 30477527 10.1186/s12984-018-0461-0 PMC6258149

[15] Laver KE, Lange B, George S, Deutsch JE, Saposnik G, Crotty M. Virtual reality for stroke rehabilitation. Cochrane Database Syst Rev. 2017;11:CD008349. 10.1002/14651858.CD008349.pub4 PMC6485957 29156493

[16] Holden MK . Virtual environments for motor rehabilitation: review. *Cyberpsychol Behav*. 2005;8:187–211 discussion 212–219. 15971970 10.1089/cpb.2005.8.187

[17] de Rooij IJM, van de Port IGL, Meijer JG. Effect of virtual reality training on balance and gait ability in patients with stroke: systematic review and meta-analysis. Phys Ther. 2016;96:1905–1918. 27174255 10.2522/ptj.20160054

[18] Corbetta D, Imeri F, Gatti R. Rehabilitation that incorporates virtual reality is more effective than standard rehabilitation for improving walking speed, balance and mobility after stroke: a systematic review. J Physiother. 2015;61:117–124. 26093805 10.1016/j.jphys.2015.05.017

[19] Darekar A, McFadyen BJ, Lamontagne A, Fung J. Efficacy of virtual reality-based intervention on balance and mobility disorders post-stroke: a scoping review. J Neuroeng Rehabil. 2015;12:46. 25957577 10.1186/s12984-015-0035-3 PMC4425869

[20] Lee HS, Park YJ, Park SW. The effects of virtual reality training on function in chronic stroke patients: a systematic review and meta-analysis. Biomed Res Int. 2019;2019:7595639. 31317037 10.1155/2019/7595639 PMC6604476

[21] Mohammadi R, Semnani AV, Mirmohammadkhani M, Grampurohit N. Effects of virtual reality compared to conventional therapy on balance poststroke: a systematic review and meta-analysis. J Stroke Cerebrovasc Dis. 2019;28:1787–1798. 31031145 10.1016/j.jstrokecerebrovasdis.2019.03.054

[22] de Rooij IJM, van de Port IGL, Visser-Meily JMA, Meijer JG. Virtual reality gait training versus non-virtual reality gait training for improving participation in subacute stroke survivors: study protocol of the ViRTAS randomized controlled trial. Trials. 2019;20:89. 30696491 10.1186/s13063-018-3165-7 PMC6352452

[23] Hatano S . Experience from a multicentre stroke register: a preliminary report. Bull World Health Organ. 1976;54:541–553. 1088404 PMC2366492

[24] Holden MK, Gill KM, Magliozzi MR, Nathan J, Piehl-Baker L. Clinical gait assessment in the neurologically impaired. Reliability and meaningfulness. Phys Ther. 1984;64:35–40. 6691052 10.1093/ptj/64.1.35

[25] van de Port IG, Wevers LE, Lindeman E, Kwakkel G. Effects of circuit training as alternative to usual physiotherapy after stroke: randomised controlled trial. BMJ. 2012;344:e2672. 22577186 10.1136/bmj.e2672 PMC3349299

[26] Post MW, van der Zee CH, Hennink J, Schafrat CG, Visser-Meily JM, van Berlekom SB. Validity of the Utrecht Scale for Evalulation of Rehabilitation-Participation. Disabil Rehabil. 2012;34:478–485. 21978031 10.3109/09638288.2011.608148

[27] van der Zee CH, Priesterbach AR, van der Dussen L, et al. Reproducibility of three self-report participation measures: the ICF Measure of Participation and Activities Screener, the participation scale, and the Utrecht Scale for Evaluation of Rehabilitation-Participation. J Rehabil Med. 2010;42:752–757. 20809057 10.2340/16501977-0589

[28] Franchignoni F, Horak F, Godi M, Nardone A, Giordano A. Using psychometric techniques to improve the Balance Evaluation Systems Test: the mini-BESTest. J Rehabil Med. 2010;42:323–331. 20461334 10.2340/16501977-0537 PMC3228839

[29] King L, Horak F. On the mini-BESTest: scoring and the reporting of total scores. Phys Ther. 2013;93:571–575. 23547173 10.2522/ptj.2013.93.4.571

[30] Punt M, van Alphen B, van de Port IG, et al. Clinimetric properties of a novel feedback device for assessing gait parameters in stroke survivors. J Neuroeng Rehabil. 2014;11:30. 24597594 10.1186/1743-0003-11-30 PMC3974014

[31] van de Port I, Punt M, Meijer JW. Walking activity and its determinants in free-living ambulatory people in a chronic phase after stroke: a cross-sectional study. Disabil Rehabil. 2020;42:636–641. 30326756 10.1080/09638288.2018.1504327

[32] Braun V, Clarke V. Using thematic analysis in psychology. Qual Res Psychol. 2006;3:77–101.

[33] van Mierlo ML, van Heugten CM, Post MW, Hajos TR, Kappelle LJ, Visser-Meily JM. Quality of life during the first two years post stroke: the Restore4Stroke cohort study. Cerebrovasc Dis. 2016;41:19–26. 26580841 10.1159/000441197

[34] Kwakkel G, Kollen B, Twisk J. Impact of time on improvement of outcome after stroke. Stroke. 2006;37:2348–2353. 16931787 10.1161/01.STR.0000238594.91938.1e

[35] Winters C, Kwakkel G, van Wegen EEH, Nijland RHM, Veerbeek JM, Meskers CGM. Moving stroke rehabilitation forward: the need to change research. NeuroRehabilitation. 2018;43:19–30. 30056434 10.3233/NRE-172393

[36] Jolkkonen J, Kwakkel G. Translational hurdles in stroke recovery studies. Transl Stroke Res. 2016;7:331–342. 27000881 10.1007/s12975-016-0461-y

[37] Stinear CM . Stroke rehabilitation research needs to be different to make a difference. F1000Res. 2016;5:1467. 10.12688/f1000research.8722.1 PMC4920210 27408689

[38] Punt M, Bruijn SM, van de Port IG, de Rooij IJM, Wittink H, van Dieen JH. Does a perturbation-based gait intervention enhance gait stability in fall-prone stroke survivors? A pilot study. J Appl Biomech. 2019;35:173–181. 30676147 10.1123/jab.2017-0282

[39] Maier M, Rubio Ballester B, Duff A, Duarte Oller E, Verschure P. Effect of specific over nonspecific VR-based rehabilitation on poststroke motor recovery: a systematic meta-analysis. Neurorehabil Neural Repair. 2019;33:112–129. 30700224 10.1177/1545968318820169 PMC6376608

[40] Kal E, Winters M, van der Kamp J, et al. Is implicit motor learning preserved after stroke? A systematic review with meta-analysis. PLoS One. 2016;11:e0166376. 27992442 10.1371/journal.pone.0166376 PMC5161313

[41] Cano Porras D, Sharon H, Inzelberg R, Ziv-Ner Y, Zeilig G, Plotnik M. Advanced virtual reality-based rehabilitation of balance and gait in clinical practice. Ther Adv Chronic Dis. 2019;10:1–16. 10.1177/2040622319868379 PMC6710712 31489154

[42] Fishbein P, Hutzler Y, Ratmansky M, Treger I, Dunsky A. A preliminary study of dual-task training using virtual reality: influence on walking and balance in chronic poststroke survivors. J Stroke Cerebrovasc Dis. 2019;28:104343. 31495668 10.1016/j.jstrokecerebrovasdis.2019.104343

[43] Wiley E, Khattab S, Tang A. Examining the effect of virtual reality therapy on cognition post-stroke: a systematic review and meta-analysis. Disabil Rehabil Assist Technol. 2020;1–11. doi: 10.1080/17483107.2020.1755376. 32363955

[44] Moreno A, Wall KJ, Thangavelu K, Craven L, Ward E, Dissanayaka NN. A systematic review of the use of virtual reality and its effects on cognition in individuals with neurocognitive disorders. Alzheimers Dement. 2019;5:834–850. 10.1016/j.trci.2019.09.016 PMC6881602 31799368

